# The wonders of anticoagulation

**DOI:** 10.3389/fcvm.2024.1517109

**Published:** 2025-01-13

**Authors:** Hugo ten Cate

**Affiliations:** ^1^ Thrombosis Expertise Center, Maastricht University Medical Centre, Maastricht, Netherlands; ^2^ CARIM School for Cardiovascular Diseases, Faculty of Health, Medicine and Life Sciences, Maastricht University, Maastricht, Netherlands; ^3^ Center for Thrombosis and Hemostasis, Gutenberg University Medical Center, Mainz, Germany

**Keywords:** vitamin K antagonists, direct oral anticoagulant, adherence–compliance–persistence, factor XI inhibitors, frailty

## Introduction

Over the past few decades, anticoagulant medication has changed dramatically. During the past century (from 1950 onward) the use of heparins and vitamin K antagonists (VKAs) became common practice for the prevention and treatment of venous thromboembolism (VTE), the prevention of stroke in atrial fibrillation (AF), and other indications (1). From the 1980s onward, the introduction of low molecular weight heparins (LMWHs) led to the rapid replacement of unfractionated heparin for the prevention and (initial) treatment of VTE (2). Subcutaneous administration of LMWHs allowed for ambulatory management of many patients (3). From the 1990s onward, the introduction of direct oral anticoagulants (DOACs, also known as “NOACs”) led to a similarly rapid replacement of VKAs for the major indications involving prolonged oral anticoagulation, VTE and AF (4, 5).

The basis for this pharmaceutical transition was established by the immense gain in knowledge of the structure and function of coagulation enzymes and their natural inhibitors. Many years of research by academics and pharmaceutical companies alike paved the way for the successful marketing of the LMWH danaparoid and the synthetic glycosaminoglycan molecule fondaparinux (6, 7). The development of DOACs was led by company-based researchers who spent many years identifying suitable small molecules with the desired specificity for their target (thrombin or factor Xa), but also with the appropriate characteristics to allow oral ingestion and adsorption from the gastrointestinal tract, and with pharmacological properties enabling once or twice daily dosing (8–10). The high quality of this research and the successful translation to clinical trials and subsequent implementation cannot be overestimated.

Large clinical trials comparing DOACs with VKAs showed non-inferior or superior efficacy and safety, compared with a VKA (warfarin), with a class effect of reduced intracranial bleeding for all DOACs (11, 12). Due to these encouraging features, coupled with strong marketing publicity, DOACs flooded the “markets”; the large volumes of patients with VTE, and particularly AF, meant big business worldwide. The downside of this success story may have been that there was little time left for reflection on possible hurdles and caveats.

### Which caveats could have been foreseen?

One of the key differences between the “old” (heparins, VKAs) and “new” anticoagulants was the move from a laboratory-guided therapy toward a fixed dose policy. For heparins, activated partial thromboplastin time-controlled therapy had been important [in cases of intravenous (IV) dosing] and for VKA, the determination of the prothrombin time [translated into International Normalized Ratio (INR)] was and remains pivotal to titrate the optimal dose for the individual patient (13, 14). Ideally, VKA management is supported by trained anticoagulation service personnel; in countries such as the Netherlands, there is still an efficient network of anticoagulation clinics (“Trombosediensten”).

With the introduction of DOACs, these anticoagulation clinics did not play any role of importance, leaving DOAC management to the prescriber and general practitioner with support from the pharmacy. In practice, my impression is that this vulnerable system of follow-up of patients on DOACs is suboptimal. My international colleagues have oftentimes suggested using the system of existing anticoagulation clinics to organize annual checks for patients on DOACs and to serve as a helpdesk for questions from patients and caregivers. However, the Dutch insurance companies have consistently deferred any serious discussions on such initiatives on the basis of the added costs of these clinics to the already expensive DOAC treatment; now that most of these drugs are or will be generic, the cost argument becomes less important. In fact, currently, one insurer actively promotes the replacement of remaining VKA treatment with DOACs as much as possible and advocates for the scaling down of anticoagulation clinics into centralized “desks” for dosing, which is bizarre as the remaining patients on VKAs are even more complex than before.

### How can DOAC management be improved?

Patients on DOACs deserve proper drug management, similar to that for drugs taken to treat diabetes, hypertension, or hypercholesterolemia. So-called cardiovascular risk management for patients with AF is a successful instrument in our country. DOAC control is only occasionally part of this system, while general knowledge of indications, mode of action, drug–drug interactions (DDI), pharmacology, and side effects is poor among patients and caregivers (DUTCH-AF study, submitted). It is probably a combination of these factors that underlies the surprising outcomes of the Dutch FRAIL-AF study in frail elderly patients with AF that showed that the conversion from VKAs to DOACs led to *more* bleeding complications as compared to continuing with VKAs, without any reduction in thromboembolic complications (15). While there is debate about several trial elements, including selection bias (only patients already on VKAs were enrolled), the outcomes should have triggered concern about the *quality* of DOAC care in this country, at this moment. Most likely, DOACs are also suitable drugs for frail patients, as shown by other studies (16), but the demonstrated inferior safety, even for hemorrhagic stroke, is alarming. A consequence must be a critical revision of our follow-up system for DOACs, focusing more on proper patient information and guidance, addressing avoidable problems such as suboptimal dosing based on inappropriate subjective arguments (frail patient, prone to falls and bleeding), but also based on a lack of insight in individual pharmacodynamics, especially in an aging population.

### A place for DOAC monitoring?

There has been much debate about the need for laboratory monitoring of DOACs and it is a pity that this discussion was not more broadly held at the introduction of these agents (17). It could be foreseen, as is recognized in most current guidelines, that at least in acute settings such as major bleeding or thromboembolism, while on DOAC therapy or in peri-procedural settings such as surgery or thrombolysis, there would be a need for determination of a DOAC plasma level, preferably even by a point-of-care (POC) device; the INR still is an undisputed biomarker for VKAs in the emergency setting. Still, the adagio remained “no monitoring necessary” for a long time. The result is that even after >15 years of DOAC use, a POC assay is still lacking, except for a rapid urine test (18).

Another assumption at the onset was that DOACs would be markedly safer, driven by the class effect of fewer intracranial bleeds. The implicit conclusion was that reversal agents would not be needed in the absence of frequent life-threatening bleeding complications, an unfortunate misconception. The positive exception was the Boehringer company which, soon after introducing dabigatran, decided to also develop a specific reversal agent; the monoclonal antibody fragment idarucizumab is a rapidly acting reversal agent without intrinsic procoagulant properties (19). Factor Xa inhibitor-producing companies decided not to invest in developing reversal agents, which may be understandable from their short-term financial outlook but not from a societal perspective where thousands of patients take a daily dose of a potentially harmful Factor Xa inhibitor without a proper antidote being available. The idea that, in a case of major bleeding, prothrombin concentrate would be sufficient has been essentially refuted by the recent Andexa-I study outcomes, which showed the superior hemostatic effect of the reversal agent Andexia for intracranial bleeding compared with prothrombin complex concentrate (20). The fact that there still is debate regarding the costs and adverse effects of Andexia is relevant and the need for refinement of this and other reversal agents is evident, but the fact that this discussion takes place while most DOACs are already out of patent illustrates the lack of careful thinking upfront when developing these potent factor Xa-targeted DOACs.

### Anticoagulation in a frail elderly population

Except for acute situations such as major bleeding, there should be further discussion and study regarding the application of DOACs in frail elderly patients, as this population is increasing among those with AF. Our recent work and that of others clearly show concerning deviations in plasma DOAC levels when compared with on-therapy ranges derived from the initial large trials (21–23). Importantly, excess plasma levels are associated with bleeding risk (24, 25). Most concerning is that it is not immediately evident what the reasons are for the deviating, especially the too-high, plasma concentrations; it is usually not directly linked to incorrect dosing or renal insufficiency. For the time being this may mean that in practice, a check of plasma levels in those over the age of 75 years or so may be worthwhile to explore whether a specific DOAC is appropriate for a given subject, certainly in cases of frailty and other potential factors such as DDI and renal impairment. While this policy may raise criticism based on the lack of proper pharmacokinetics when sampling single blood draws and the inappropriate use of on-therapy ranges and so forth, common sense may suffice to at least estimate whether the DOAC used is reasonable to begin with, should be replaced by another, or be tailored in dose within registered ranges. Obviously, clinical trials need to address the potential utility of DOAC laboratory monitoring for establishing long-term safety among frail DOAC users. Finally, one should be aware of VKAs as a more than reasonable alternative.

### VKAs remain a proper alternative for DOACs

Many starting physicians will have hardly any experience with VKAs, so my observation is that this alternative to DOACs is hardly ever considered in practice. Many think that VKAs are old-fashioned (correct), complex (partially correct), and dangerous (incorrect, at least not much worse than DOACs). However, important indications for VKAs remain in place as DOACs were inferior in patients with mechanical heart valves, antiphospholipid antibody syndrome (at least in those with triple-positive antibodies), and moderate-to-severe mitral valve stenosis-associated AF (26). Moreover, the practical advantage of VKAs is the managed care, which, when organized properly, provides individual tailoring that considers all possible factors including DDI and renal impairment. For patients with anticipated poor drug adherence, this may also be an advantage of VKAs over DOACs. VKAs have the disadvantage of increased vascular calcification and perhaps a negative impact on renal function in those with renal insufficiency (27, 28). The surprising advantages of VKAs may be increased survival in subjects with cancer (which may be due to the inhibition of specific proteins such as Gas-6) (29) and in patients with AF, at least while on phenprocoumon, the single VKA in Germany (partially explained) (30); when compared to Factor Xa inhibitors, patients on dabigatran had a comparable survival advantage. This possible survival benefit, while prone to bias, sheds new light on this old class of agents and may be reassuring for those who feared that VKAs, when indicated, would cause more harm (e.g., vascular calcification) than benefit. Altogether, one should not discard VKAs as a treatment alternative even in frail elderly with AF, provided that a good time in the therapeutic range is achieved; the latter is best obtained with phenprocoumon, but this medication is not available in many countries, unfortunately. The German colleagues appropriately call for a randomized trial to assess the merits of phenprocoumon compared with DOACs (30).

### A place for new anticoagulants?

Finally, following the initial enthusiasm about DOACs, we are entering a period of greater realism regarding the limitations in the safety of DOACs (and VKA), with a remaining annual risk of major bleeding of at least 2%–3% on average, and substantial variation among individuals that is hard to predict with current risk scores.

The hope among pharmaceutical companies (and their investors) is that safety can be further enhanced by addressing other targets, including factor XIa (31). On the one hand, this wishful thinking stems from observational data from patients with congenital factor XI deficiency showing that it is associated with a low risk of spontaneous bleeding, in contrast to other hemophilias. On the other hand, epidemiological, genetic, and experimental evidence indicates that FXI is associated with thrombus formation and is specifically linked to cardioembolic stroke and VTE. Data on atherothrombosis remain controversial (32–34).

A proof of principle human study showed that FXI gene silencing with siRNA technology markedly reduced FX levels in blood and also achieved a substantial reduction in postoperative venous thrombosis in knee replacement surgery (35). A comparable efficacy was shown for other approaches, including monoclonal antibodies and the small oral molecule milvexian (36). Based on these studies, phase 2 trials were designed in patients with VTE, but also with arterial vascular disease, including AF, acute stroke, and acute coronary syndrome (ACS). Last year, the outcomes of several phase 2 trials were published. The data unequivocally show reduced bleeding risk compared with apixaban, or rivaroxaban in the AF studies, and acceptable bleeding rates in patients with ACS or stroke [discussed in (37–39)].

However, none of these studies provided a clear conclusion about efficacy, which although not accounted for in phase 2, would have been of interest. Driven by optimism, phase 3 was initiated with studies in AF, the OCEANIC-AF and LIBREXIA-AF, with asundexian and milvexian, respectively (40, 41). While the LIBREXIA-AF trial is still ongoing, the OCEANIC-AF trial was stopped after the inclusion of close to 15,000 patients due to excess ischemic strokes in the asundexian arm. The main paper was recently published, discussing the potential reasons for failure (40). These included too-low dosing (although in plasma samples there was well over 90% inhibition of FXIa) and escape mechanisms that were not alluded to but may include bypass activation of FIX by kallikrein (42). Finally, the authors noted that the background population may have markedly changed with a lower AF burden, explaining the overall low rate of embolic stroke. A final option, that FXI activation may not be so relevant in *all* subjects with AF, was not mentioned. All the arguments shed doubt on the design of such large studies, where the contribution of FXI to thrombosis risk, the impact of the drug (dose), and possible escape mechanisms, including the kallikrein-driven activation of FIX bypassing FXI, were apparently not sufficiently explored or considered. Unfortunately, the negative trial outcome may lead to skepticism regarding the concept of FXI inhibition that may be preliminary and unjustified in the absence of mechanistic data.

## Conclusion and perspective

Although anticoagulant treatment has improved markedly from a practical perspective, its safety remains in question as the follow-up of patients on DOACs is insufficient. This certainly concerns frail elderly with AF where VKAs may even be better than DOACs if VKA care is well-managed. Attention must be paid to improving the quality of DOAC treatment, including adherence to therapy, which may also require occasional assessment of DOAC plasma level in frail patients to assess the suitability of the drug and dose. When moving forward with novel anticoagulants, e.g., factor XIa inhibitors, one should not rely on wishful thinking but carefully consider disease-related thrombosis mechanisms in diverse patients to be better prepared for and hopefully avoid more large study failures.
